# Supplementary material for: Unveiling the Bioactive Potential of the Invasive Jellyfish Phyllorhiza punctata Through Integrative Transcriptomic and Proteomic Analyses
Source: Biomolecules. 2025 Aug 4;15(8):1121. doi: 10.3390/biom15081121 (PMC12383608; doi:10.3390/biom15081121)
Supplement: Supplementary file 1 [file biomolecules-15-01121-s001.zip › Suplementary Material/FileS3/PEPFOLD4/XP_065062240_2760/clusters.html]

 PEP-FOLD clusters


Download archive of all models  here   

|  |  |  |
| --- | --- | --- |
| # | file | sOPEP |
| # cluster 1 - Size: 28 |  |  |
|  | XP\_065062240\_2760-model1 | -9.37977 |
|  | XP\_065062240\_2760-model1.1 | -9.37977 |
|  | XP\_065062240\_2760-model1.2 | -9.37977 |
|  | XP\_065062240\_2760-model1.3 | -9.37977 |
|  | XP\_065062240\_2760-model1.4 | -9.37977 |
|  | XP\_065062240\_2760-model1.5 | -9.37977 |
|  | XP\_065062240\_2760-model1.6 | -9.37977 |
|  | XP\_065062240\_2760-model1.7 | -9.37977 |
|  | XP\_065062240\_2760-model1.8 | -9.37356 |
|  | XP\_065062240\_2760-model1.9 | -9.37356 |
|  | XP\_065062240\_2760-model1.10 | -9.37356 |
|  | XP\_065062240\_2760-model1.11 | -9.37356 |
|  | XP\_065062240\_2760-model1.12 | -9.36575 |
|  | XP\_065062240\_2760-model1.13 | -9.36575 |
|  | XP\_065062240\_2760-model1.14 | -9.36575 |
|  | XP\_065062240\_2760-model1.15 | -9.36575 |
|  | XP\_065062240\_2760-model1.16 | -9.36575 |
|  | XP\_065062240\_2760-model1.17 | -9.36575 |
|  | XP\_065062240\_2760-model1.18 | -9.36575 |
|  | XP\_065062240\_2760-model1.19 | -9.36575 |
|  | XP\_065062240\_2760-model1.20 | -9.36024 |
|  | XP\_065062240\_2760-model1.21 | -9.36024 |
|  | XP\_065062240\_2760-model1.22 | -9.36024 |
|  | XP\_065062240\_2760-model1.23 | -9.36024 |
|  | XP\_065062240\_2760-model1.24 | -9.29596 |
|  | XP\_065062240\_2760-model1.25 | -9.29138 |
|  | XP\_065062240\_2760-model1.26 | -9.29138 |
|  | XP\_065062240\_2760-model1.27 | -8.71071 |
| # cluster 2 - Size: 54 |  |  |
|  | XP\_065062240\_2760-model2 | -5.44229 |
|  | XP\_065062240\_2760-model2.1 | -5.44229 |
|  | XP\_065062240\_2760-model2.2 | -5.44229 |
|  | XP\_065062240\_2760-model2.3 | -5.44229 |
|  | XP\_065062240\_2760-model2.4 | -5.44229 |
|  | XP\_065062240\_2760-model2.5 | -5.44229 |
|  | XP\_065062240\_2760-model2.6 | -5.44229 |
|  | XP\_065062240\_2760-model2.7 | -5.44229 |
|  | XP\_065062240\_2760-model2.8 | -5.39902 |
|  | XP\_065062240\_2760-model2.9 | -5.39902 |
|  | XP\_065062240\_2760-model2.10 | -5.3644 |
|  | XP\_065062240\_2760-model2.11 | -5.04963 |
|  | XP\_065062240\_2760-model2.12 | -5.00454 |
|  | XP\_065062240\_2760-model2.13 | -5.00454 |
|  | XP\_065062240\_2760-model2.14 | -4.97811 |
|  | XP\_065062240\_2760-model2.15 | -4.97811 |
|  | XP\_065062240\_2760-model2.16 | -4.95369 |
|  | XP\_065062240\_2760-model2.17 | -4.95369 |
|  | XP\_065062240\_2760-model2.18 | -4.94239 |
|  | XP\_065062240\_2760-model2.19 | -4.94239 |
|  | XP\_065062240\_2760-model2.20 | -4.94239 |
|  | XP\_065062240\_2760-model2.21 | -4.89861 |
|  | XP\_065062240\_2760-model2.22 | -4.89861 |
|  | XP\_065062240\_2760-model2.23 | -4.89861 |
|  | XP\_065062240\_2760-model2.24 | -4.8965 |
|  | XP\_065062240\_2760-model2.25 | -4.87949 |
|  | XP\_065062240\_2760-model2.26 | -4.87765 |
|  | XP\_065062240\_2760-model2.27 | -4.87765 |
|  | XP\_065062240\_2760-model2.28 | -4.87765 |
|  | XP\_065062240\_2760-model2.29 | -4.87765 |
|  | XP\_065062240\_2760-model2.30 | -4.87765 |
|  | XP\_065062240\_2760-model2.31 | -4.87765 |
|  | XP\_065062240\_2760-model2.32 | -4.87765 |
|  | XP\_065062240\_2760-model2.33 | -4.87765 |
|  | XP\_065062240\_2760-model2.34 | -4.87765 |
|  | XP\_065062240\_2760-model2.35 | -4.87765 |
|  | XP\_065062240\_2760-model2.36 | -4.87765 |
|  | XP\_065062240\_2760-model2.37 | -4.87765 |
|  | XP\_065062240\_2760-model2.38 | -4.87765 |
|  | XP\_065062240\_2760-model2.39 | -4.87765 |
|  | XP\_065062240\_2760-model2.40 | -4.86377 |
|  | XP\_065062240\_2760-model2.41 | -4.86377 |
|  | XP\_065062240\_2760-model2.42 | -4.86377 |
|  | XP\_065062240\_2760-model2.43 | -4.86377 |
|  | XP\_065062240\_2760-model2.44 | -4.86377 |
|  | XP\_065062240\_2760-model2.45 | -4.8591 |
|  | XP\_065062240\_2760-model2.46 | -4.83594 |
|  | XP\_065062240\_2760-model2.47 | -4.8071 |
|  | XP\_065062240\_2760-model2.48 | -4.80387 |
|  | XP\_065062240\_2760-model2.49 | -4.80387 |
|  | XP\_065062240\_2760-model2.50 | -4.75476 |
|  | XP\_065062240\_2760-model2.51 | -4.73122 |
|  | XP\_065062240\_2760-model2.52 | -4.71512 |
|  | XP\_065062240\_2760-model2.53 | -4.68412 |
| # cluster 3 - Size: 1 |  |  |
|  | XP\_065062240\_2760-model3 | -4.79951 |
| # cluster 4 - Size: 1 |  |  |
|  | XP\_065062240\_2760-model4 | -4.69748 |
| # cluster 5 - Size: 3 |  |  |
|  | XP\_065062240\_2760-model5 | -4.66649 |
|  | XP\_065062240\_2760-model5.1 | -4.54782 |
|  | XP\_065062240\_2760-model5.2 | -4.53043 |
| # cluster 6 - Size: 3 |  |  |
|  | XP\_065062240\_2760-model6 | -4.65894 |
|  | XP\_065062240\_2760-model6.1 | -4.6049 |
|  | XP\_065062240\_2760-model6.2 | -4.57006 |
| # cluster 7 - Size: 3 |  |  |
|  | XP\_065062240\_2760-model7 | -4.60174 |
|  | XP\_065062240\_2760-model7.1 | -4.48363 |
|  | XP\_065062240\_2760-model7.2 | -4.46628 |
| # cluster 8 - Size: 7 |  |  |
|  | XP\_065062240\_2760-model8 | -4.58736 |
|  | XP\_065062240\_2760-model8.1 | -4.58017 |
|  | XP\_065062240\_2760-model8.2 | -4.54196 |
|  | XP\_065062240\_2760-model8.3 | -4.53777 |
|  | XP\_065062240\_2760-model8.4 | -4.53056 |
|  | XP\_065062240\_2760-model8.5 | -4.52799 |
|  | XP\_065062240\_2760-model8.6 | -4.51421 |
|  |  |  |
